# Developing and Implementing an mHealth Heart Failure Self-care Program to Reduce Readmissions: Randomized Controlled Trial

**DOI:** 10.2196/33286

**Published:** 2022-03-21

**Authors:** Amber E Johnson, Shuvodra Routh, Christy N Taylor, Meagan Leopold, Kathryn Beatty, Dennis M McNamara, Esa M Davis

**Affiliations:** 1 Division of Cardiology Department of Medicine University of Pittsburgh Pittsburgh, PA United States; 2 UPMC Heart and Vascular Institute Pittsburgh, PA United States; 3 Department of Internal Medicine UPMC Pittsburgh, PA United States; 4 Department of Medicine Massachusetts General Hospital Harvard Medical School Boston, MA United States; 5 UPMC Community Provider Services Pittsburgh, PA United States; 6 Innovative Homecare Solutions of UPMC Pittsburgh, PA United States; 7 Division of General Internal Medicine Department of Medicine University of Pittsburgh Pittsburgh, PA United States

**Keywords:** mHealth, heart failure, self-care, remote monitoring, telehealth, cardiology, hospital readmission, self-management, mobile health, patient-centered

## Abstract

**Background:**

Patients admitted with decompensated heart failure (HF) are at risk for hospital readmission and poor quality of life during the discharge period. Lifestyle behavior modifications that promote the self-management of chronic cardiac diseases have been associated with an improved quality of life. However, whether a mobile health (mHealth) program can assist patients in the self-management of HF during the acute posthospital discharge period is unknown.

**Objective:**

We aimed to develop an mHealth program designed to enhance patients’ self-management of HF by increasing knowledge, self-efficacy, and symptom detection. We hypothesized that patients hospitalized with HF would be willing to use a feasibly deployed mHealth program after their hospital discharge.

**Methods:**

We employed a patient-centered outcomes research methodology to design a stakeholder-informed mHealth program. Adult patients with HF admitted to a large academic hospital were enrolled and randomized to receive the mHealth intervention versus usual care. Our feasibility outcomes included ease of program deployment, use of the clinical escalation process, duration of participant recruitment, and participant attrition. Surveys assessing the demographics and clinical characteristics of HF were measured at baseline and at 30 and 90 days after discharge.

**Results:**

The study period was between July 1, 2019, and April 7, 2020. The mean cohort (N=31) age was 60.4 (range 22-85) years. Over half of the participants were men (n=18, 58%) and 77% (n=24) were White. There were no significant differences in baseline measures. We determined that an educational mHealth program tailored for patients with HF is feasibly deployed and acceptable by patients. Though not significant, we found notable trends including a higher mean quality of life at 30 days posthospitalization among program users and a longer duration before rehospitalization, which are suggestive of better HF prognosis.

**Conclusions:**

Our mHealth tool should be further assessed in a larger comparative effectiveness trial. Our pilot intervention offers promise as an innovative means to help HF patients lead healthy, independent lives. These preliminary data suggest that patient-centered mHealth tools can enable high-risk patients to play a role in the management of their HF after discharge.

**Trial Registration:**

ClinicalTrials.gov NCT03982017; https://clinicaltrials.gov/ct2/show/NCT03982017

## Introduction

Patients hospitalized with decompensated heart failure (HF) are at risk for poor quality of life during the postdischarge period, hospital readmission within 30 days, and increased mortality [1,2]. Patient self-management of HF promotes better quality of life and longevity [3]. Preventing hospital readmission for HF is a mutual goal for patients, providers, and payers [2]. Chronic disease self-management requires patients to have self-efficacy, the knowledge to detect symptoms, and any necessary skills and tools [4,5]. Studies have shown that patient self-efficacy and symptom recognition are essential components of managing a chronic condition such as HF [3,6,7]. Additionally, interventions that develop self-management skills can improve patient knowledge and foster healthy lifestyle behaviors that are associated with improvements in quality of life [8].

Mobile health (mHealth) smartphone programs are facilitating the direct delivery of health information to patients [9]. In response, patients are becoming more knowledgeable about their conditions and are being equipped with the skills to care for themselves [9,10]. Nevertheless, the utility of mHealth for self-management among patients with HF is still under investigation. The older age of most patients with HF has been considered a barrier to the adaptation of smartphone technologies in this patient population [11]. Additionally, patients with HF may lack the physical capacity to follow an mHealth program during an exacerbation of their illness. On the contrary, research has demonstrated that patients with acute HF are at the highest risk for adverse outcomes and thus have the greatest need for additional support during the period after HF hospitalization [12]. Pragmatic research is needed to identify feasible mHealth methods for HF patients to care for themselves, especially after hospitalization.

Whether a smartphone program can assist patients in HF self-management during the immediate posthospital period within 30 days is unknown. Moreover, to design future comparative effectiveness trials, it is important to demonstrate that patients admitted with HF can be enrolled in such studies at discharge. Although other mHealth programs have been created for patients with stable HF [13], our goal was to develop an mHealth self-management program designed to enhance patient HF knowledge, self-efficacy, and symptom perception to prevent repeat exacerbation. We hypothesized that patients hospitalized with HF would be willing to use a feasible mHealth program.

## Methods

### Setting and Participants

The University of Pittsburgh Medical Center (UPMC) Presbyterian Hospital is a 900-bed academic hospital with inpatient cardiology and HF services. We included adult patients aged 18 years and older who were admitted to UPMC Presbyterian Hospital with acute decompensated HF as determined by a documented admission history. Patients with either systolic or diastolic left ventricular HF and who had a personal smartphone were eligible. We excluded patients with end-stage HF (eg, receipt of heart transplant, listed or under evaluation for heart transplant, inotrope dependence, under hospice care, and had a ventricular assist device or under evaluation for a ventricular assist device). Patients were excluded if they were discharged to a nursing home or were participants in other telemonitoring programs.

### Ethical Considerations

This project was approved by the University of Pittsburgh Institutional Review Board and registered with National Clinical Trials (NCT03982017). Patients were enrolled and randomized after providing written informed consent. Participants were compensated US $40 upon study completion.

### Heart Failure Self-care Mobile App to Reduce Readmissions Trial (HF-SMART) Program

**Figure 1:**
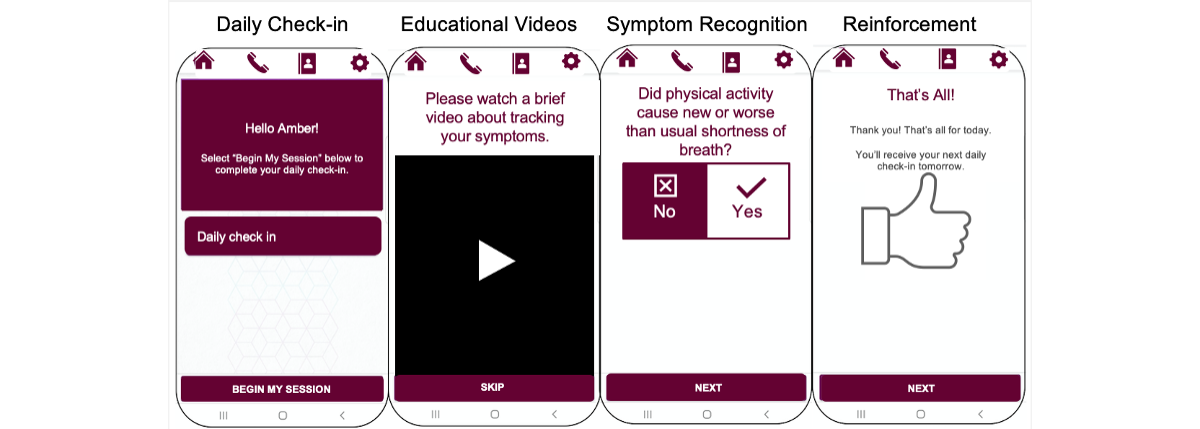
Depiction of the Heart Failure Self-care Mobile Application to Reduce Readmissions Trial (HF-SMART) program.

We employed a patient-centered outcomes research (PCOR) methodology to design a stakeholder-informed program [14]. Key stakeholders comprised patients, physicians, nurses, platform developers, and patient education and mHealth experts. The intervention was designed to complement the care patients would typically receive at a posthospital follow-up cardiology visit. The mHealth program featured a patient-facing, internet-based platform for use on any smartphone and was designed in compliance with industry standards for mHealth programs [15]. The program consisted of a secure website with content tailored to patients with chronic HF, including educational videos and daily prompts, as can be seen in Figure 1. Patients were instructed to navigate to their unique link to access the program content [16]. Other key features included alerts that directed patients to contact medical personnel‎ in the event of urgent health issues, active monitoring of patient HF data by nurses, interactive feedback of patients’ symptom assessment with biometric tracking, and reminders for medication adherence.

### Study Design

This pragmatic feasibility study included patients with acute exacerbations of chronic HF with either reduced or preserved ejection fraction. Our primary objective was to determine the feasibility of developing, implementing, and assessing the HF-SMART mHealth program in patients with HF at hospital discharge compared with usual care. We assessed the following feasibility criteria: duration of participant recruitment, participant attrition, participant acceptability, ease of program deployment, and necessity of clinical escalation for participants requiring medical attention. Our secondary outcome was to determine patient satisfaction with the mHealth program.

Using a random number generator, the research coordinator randomized participants in a simple, unblinded 1:1 fashion to either the intervention arm—mHealth program plus usual HF care—or the usual care arm. At our institution, usual care consists of routine discharge planning that includes a review of the discharge medications and clinical discharge summary with the recommended follow-up appointments with the nurse. Occasionally, follow-up appointments are scheduled before discharge; however, more often, patients are responsible for scheduling their own appointments. At the discretion of the discharging provider, some patients may receive standard educational materials on their illness and postdischarge care. For example, the electronic medical record contains a congestive heart failure patient handout that can be printed for the patient at the time of discharge.

### Survey Measures

A research assistant administered the baseline and demographic survey instruments at the bedside. Follow-up quality of life surveys were conducted via telephone at 30 and 90 days postdischarge. Survey measures were selected based on their validity in medical populations and their established psychometric properties. For those randomized to the intervention arm, we also measured postintervention participant satisfaction using a proprietary assessment (see Multimedia Appendix 1) with elements from other usability questionnaires [17,18], thus meeting face validity.

At baseline, all participants completed the 10-item Patient Activation Measure (PAM) [6,7], the 10-item Perceived Stress Scale [19], the 2-item Patient Health Questionnaire [20], and the 13-item Social Network Index [21]. Each of these measures has been shown to be associated with HF self-management and clinical cardiovascular outcomes [22-25]. Patient activation, a measure that incorporates self-efficacy, knowledge, and engagement, was measured with the PAM. The 13-item version of the PAM demonstrates good internal consistency overall (Cronbach *α*=.81) [6]. Higher scores on the PAM indicate more self-efficacy, knowledge, and engagement. The Perceived Stress Scale (higher score denotes greater stress), Patient Health Questionnaire (a score of 3 or higher denotes depression), and Social Network Index (higher score denotes greater social support) were included in the comprehensive baseline assessments of our participants to determine the psychological aspects known to be associated with cardiovascular outcomes [24,26,27]. Research examining the role of social support in chronic disease self-management indicates that social support improves adherence to medications and dietary regimens and lessens patient-reported depression [28].

### Outcome Variables

Our independent variable was receipt of the mHealth program (yes or no). The primary outcome was feasibility. Our secondary outcome was patient satisfaction. We assessed the following exploratory outcome variables. Readmission was defined as a nonelective hospital admission via the emergency department, directly from the outpatient or residential setting, or transfer from another health system within 30 or 90 days. We dichotomized readmission (yes or no) and measured time to readmission as a continuous variable. Death was measured if it occurred within the study time frame.

Quality of life was measured with the Kansas City Cardiomyopathy Questionnaire (KCCQ) at 30 and 90 days postindex hospital discharge. The KCCQ consists of 23 questions that use a Likert scale and features an intraclass correlation coefficient of 0.88 [29]. Subscales consist of physical limitation, symptom frequency, quality of life, and social limitation scores. A higher score on the KCCQ indicates a higher quality of life.

Covariates included age, self-reported race and ethnicity, binary (male/female) sex category, left ventricular ejection fraction, relevant laboratory results at the time of admission, clinically relevant comorbid conditions, and number of medications at the time of discharge.

### Statistical Analysis

Although our study was not powered to detect statistical differences between groups, we conducted exploratory analyses on the available data. We used chi-square (or Fisher exact test) to assess if the intervention was associated with reduced readmission. We also used *t* tests to compare the mean time to readmission by intervention arm; time to readmission by race, sex, and systolic versus diastolic HF; and baseline PAM by race, sex, systolic versus diastolic HF.

## Results

### Setting and Participants

**Figure 2:**
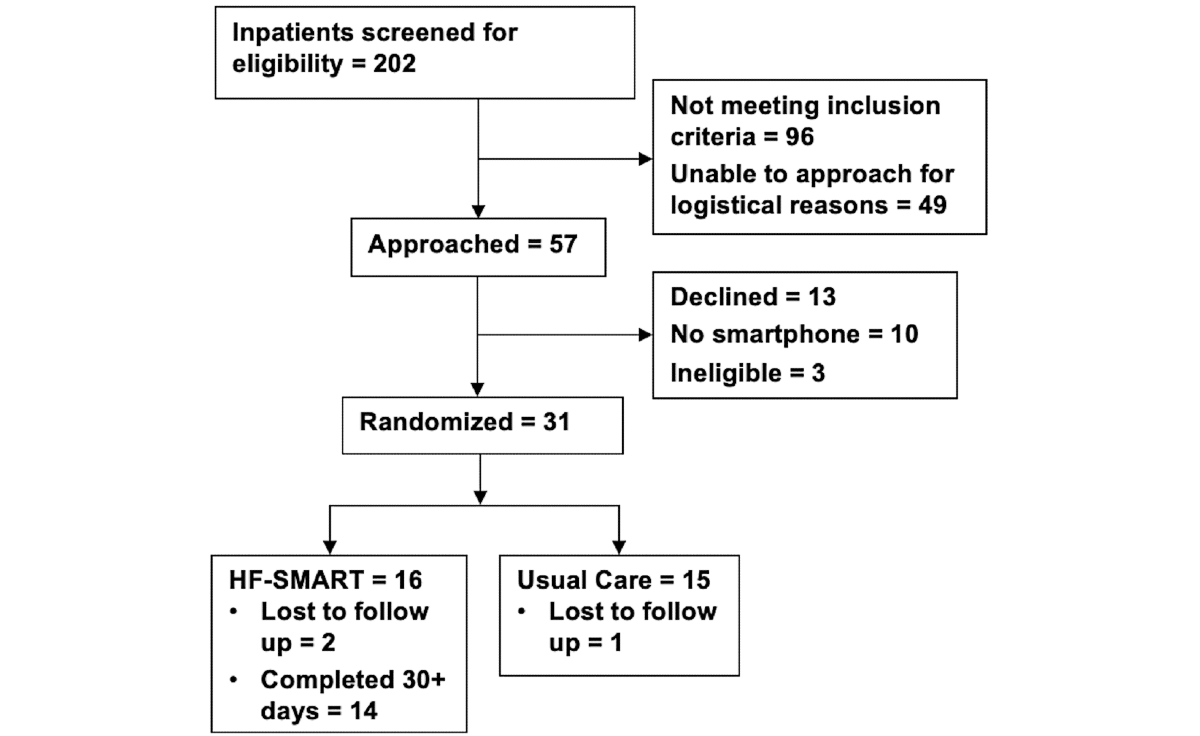
Patient flow diagram for the study.

The study period was from July 1, 2019, to April 7, 2020. Participants were enrolled for a median of 2.2 (range 0.8-3) months with additional clinical assessments of up to 90 days. Unfortunately, the study was terminated prior to enrolling our prespecified goal of 50 participants due to institutional restrictions on clinical research secondary to the SARS-CoV-2 (COVID-19) pandemic. Figure 2 shows the patient flow for the study. Many patients were excluded from the study because they were discharged to a skilled nursing facility or with home care services. The largest barrier to recruitment was reaching, consenting, and enrolling the patients at their bedsides during the acute hospital stay. Nevertheless, 31 of the 57 (54.4%) patients that were approached agreed to participate, of which 16 were assigned to receive the program.

### Heart Failure Self-care Mobile Application to Reduce Readmissions Trial (HF-SMART) Program

The program deployment was uneventful with no need for clinical escalation of care, and there were no reported systems failures by participants, signifying that the website worked as intended. Retention in the program was satisfactory, with 14 of 16 (87.5%) participants completing at least 30 days of the program. Patient satisfaction was high, with all surveyed participants agreeing with the statements, “Overall, I am satisfied with my experience in the HF-SMART program” and “I would recommend this program to others.”

### Survey Measures

Participant demographics and baseline clinical data are shown in Table 1. Groups were clinically similar based on laboratory data at the time of admission, type of HF (preserved vs reduced ejection fraction), and number of medications at discharge. The mean age was 60.4 (range 22-85) years. Over half of participants were men (n=18, 58%) and 77% (n=24) were White. There were no significant differences in baseline health psychology measures as indicated by the Patient Activation Measure, Perceived Stress Scale, Patient Health Questionnaire, and Social Network Index.

**Table 1:** Baseline characteristics of the cohort.

	HF-SMART^a^	Usual care	*P* value
Patients, n	16	15	N/A^b^
Follow-up (days), mean (SD)	62.4 (23.4)	64.8 (24.9)	.78
Age (years) mean (SD)	60.1 (12.9)	60.7 (15.0)	.91
Male sex, n (%)	10 (62.5)	8 (53.3)	.61
White race, n (%)	12 (75)	12 (80)	.99
Hispanic, n (%)	2 (12.5)	0 (0)	.48
HF^c^ with reduced ejection fraction, n (%)	10 (62.5)	7 (46.7)	.38
Positive admission troponin, n (%)	3 (18.8)	4 (26.7)	.99
Admission BNP^d^, mean (SD)	623 (562.3)	739 (542.3)	.61
Admission hemoglobin, mean (SD)	12.2 (2.2)	12.5 (1.71)	.66
Admission creatinine, mean (SD)	1.4 (0.6)	1.3 (0.6)	.65
Number of discharge medications, mean (SD)	13.8 (5.2)	14.2 (7.2)	.84
Patient Activation Measure [5,6], mean (SD)	64.4 (12.3)	60.8 (14.5)	.48
Perceived Stress Scale [19], mean (SD)	18.1 (8.4)	17.7 (10.6)	.91
Positive Patient Health Questionnaire [30] Screen, n (%)	5 (31.3)	4 (26.7)	.99
Social Network Index [21], mean (SD)	5.3 (1.9)	6 (2.0)	.34

^a^HF-SMART: Heart Failure Self-care Mobile App to Reduce Readmissions Trial.

^b^N/A: not applicable.

^c^HF: heart failure.

^d^BNP: b-type naturetic protein

### Outcome Variables

We collected longitudinal data for 29 of the initial 31 participants. Table 2 shows the clinical outcomes of treatment arms with respect to readmission, quality of life, and mortality. At 30 days, KCCQ scores were higher among the 6 participants who responded to the telephone survey in the intervention arm. We found that 5 patients were readmitted within 30 days for a readmission rate of 16.7% (n=3, 18.8% in the intervention group vs n=2, 13.3% in the usual care group). We also found that 9 patients were readmitted within 90 days for a readmission rate of 30% (n=5, 31.3% in the intervention group vs n=4, 28.6% in the usual care group). Only one person died during the study period and was in the usual care group. None of these differences were statistically significant.

**Table 2:** Characteristics of treatment arms with respect to readmission, quality of life, and mortality.

	HF-SMART^a^ (n=16)	Usual care (n=14)	*P* value
Readmitted within 30 days, n (%)	3 (18.8)	2 (13.3)	.65
Readmitted within 90 days, n (%)	5 (31.3)	4 (28.6)	.70
KCCQ^b^ at 30 days, n (%)	6 (37.5)	9 (64.3)	N/A^c^
KCCQ score at 30 days, mean (SD)	88.57 (15.89)	63.06 (31.34)	.09
KCCQ at 90 days, n (%)	3 (18.8)	3 (21.4)	N/A
KCCQ score at 90 days, mean (SD)	71.53 (16.83)	94.1 (7.69)	.10
Death, n (%)	0 (0)	1 (7.1)	N/A

^a^HF-SMART: Heart Failure Self-care Mobile App to Reduce Readmissions Trial.

^b^KCCQ: Kansas City Cardiomyopathy Questionnaire.

^c^N/A: not applicable.

## Discussion

### Principal Results

We determined that an mHealth self-management program for HF patients is feasible and acceptable to patients. This pilot study was designed as a prospective, randomized controlled trial to assess the feasibility of deploying the intervention in our patient population. We also assessed patient outcomes in terms of readmission and quality of life. However, these clinical end points could not be assessed in a statistically meaningful way. Nevertheless, we found a higher mean quality of life among program users at 30 days and longer duration before readmission, which are suggestive of better HF management using a customary 30-day end point [31]. On the contrary, the mean KCCQ score at 90 days posthospitalization was higher in the usual care group despite high patient satisfaction, but the reliability of the 90-day findings is limited by the very small sample size. We infer from these encouraging data that our mHealth program has promise in further testing in a larger trial. These data provide evidence that patient-centered mHealth programs have a role in the management of high-risk HF patient populations. Our pilot intervention is a favorable and innovative tool to help HF patients lead healthier lives.

### Comparison With Prior Work

Professional guidelines encourage short-interval follow-up (eg, less than 1 week) of HF patients once discharged from the hospital [1]. The posthospitalization period can be difficult for patients recovering from acute HF exacerbations due to the little support available to them as they transition from hospital to home and the limited in-person follow-up cardiology appointments [32]. Prior mHealth interventions, though successful at producing their prespecified clinical outcomes, have been heavily reliant on physician expertise to manage patients remotely [33,34]. Others have shown that interventions including community health workers can provide a support system for the HF patient population [35]. However, these prior efforts are resource intensive and require coordination of clinicians’ and patients’ time. During the COVID-19 pandemic, face-to-face interactions with clinicians and community health workers would have violated physical distancing recommendations, thus increasing the risk of COVID-19 infections, complications, or death. Patient-centered mHealth technologies offer a practical alternative to interpersonal interventions [36]. Our mHealth intervention was designed to complement the care patients would typically receive at an in-person clinic visit. The posthospitalization period has many obstacles for patients with HF, and self-management mHealth tools that increase self-efficacy and skills to implement the HF treatment plan by the cardiologist may reduce readmissions.

We add to the existing literature that the recruitment, enrollment, and implementation of a smartphone-based self-management intervention can be accomplished among patients with acute HF exacerbations. This timing of enrollment just prior to hospital discharge is critical for patients to take charge of their health and commit to self-management activities. Furthermore, the utility of enrolling inpatients not only proved feasible, but we also found that those recruited remained engaged in the intervention. These findings will be useful for future larger trials of similar interventions.

Researchers have described the potential barriers to the deployment of mHealth and the uptake of digital device use in an older patient population [37]. We found that lack of the requisite technology was not a reason for older patients to decline participation in this study. At the time of enrollment, all participants had smartphones and were motivated to use an HF self-care program. As the reliance on technological advancement for the provision of health services increases, there is growing support for underserved communities to receive equitable access to the necessary technological tools. These include mHealth technology, digital devices, and broadband internet for older adults [38]. Our findings should inspire future research on mHealth efficacy among older patient populations with chronic conditions.

Although patient engagement with mHealth, personalized medical technology, and patient-facing applications continues to grow, patient-centered mHealth has yet to be optimized [39]. The COVID-19 pandemic has facilitated a paradigm shift toward embracing telehealth technologies and empowering patients to manage more of their own care. Patients’ experiences, skills, activation, and other unique contextual factors are all important to consider when developing mobile interventions for patients with HF [40]. 

In our cohort of high-risk patients with HF, we aimed to explore how psychosocial aspects at the time of HF hospitalization affected readmission and use of our mHealth program. Participants did not differ on baseline psychometric measures, and survey responses were not predictive of clinical outcomes, though underpowered to detect significance. Limitations of prior studies include no comparison group [13] and evaluations of only relatively low-risk, ambulatory cohorts [13,34]. Prior studies also evaluated the change in quality of life compared with baseline [13]. We did not assess quality of life at baseline because our patients were admitted to the hospital at the time of baseline assessment and because the KCCQ requires the patient to recall how they felt in the 2 weeks preceding the questionnaire. Being ill from acute decompensated HF likely decreases quality of life and biases a patient’s response. Thus, an improvement in KCCQ above such a baseline is unlikely to be reflective of the intervention, rather reflecting the patient having recovered from their acute HF exacerbation.

### Limitations and Strengths

There are several important limitations to this study. First, due to slow enrollment, our cohort was small. We planned to modify the study so that all participants would receive the intervention to further assess the intervention’s effect; however, the study was terminated prior to modification due to restrictions related to the COVID-19 pandemic. Nevertheless, experts have suggested that pilot feasibility studies with a sample size of at least 12 participants provide valuable preliminary information when planning subsequent effectiveness trials [41]. Second, longitudinal data on health care utilization and medication adherence were not collected. Lastly, we were unable to determine the effect of inequitable access to technological advances in underserved communities due to the limited assessment of participant sociodemographic information and because smartphone ownership was an inclusion criterion. Despite these limitations, this study has several strengths. Our intervention had low attrition and several objectively measured positive outcomes for cardiovascular health, which are reflective of our formative work and prioritization of patient self-management. Another strength is that the program was developed with validated PCOR methods including stakeholder engagement to create a patient-centered program that increases the likelihood of the intervention being acceptable [14].

### Conclusions

In conclusion, our pilot study showed that an educational HF self-management mHealth program was feasibly deployed and the patient experience was positive. Although we showed a trend toward a better 30-day quality of life, this study was not powered to detect differences between arms on account of early termination due to clinical research restrictions resulting from the COVID-19 pandemic. Nevertheless, we demonstrated that enhanced HF self-management is welcomed by patients and shows promise to improve quality of life posthospitalization. By demonstrating the proof of concept, this pilot study warrants further evaluation in a larger and more diverse cohort. Furthermore, this mHealth HF program can be modified to assist with the management of other chronic diseases.

## Appendix

Multimedia Appendix 1 Usability survey.

Multimedia Appendix 2 CONSORT eHEALTH checklist (V 1.6.1).

## Abbreviations

HF: heart failure
HF-SMART: Heart Failure Self-care Mobile Application to Reduce Readmissions Trial
KCCQ: Kansas City Cardiomyopathy Questionnaire
mHealth: mobile health
PAM: Patient Activation Measure
PCOR: Patient-Centered Outcomes Research
UPMC: University of Pittsburgh Medical Center
USPSTF: United States Preventive Services Task Force
