# Supplementary material for: Developing and Implementing an mHealth Heart Failure Self-care Program to Reduce Readmissions: Randomized Controlled Trial
Source: JMIR Cardio. 2022 Mar 21;6(1):e33286. doi: 10.2196/33286 (PMC8981015; doi:10.2196/33286)
Supplement: Multimedia Appendix 1 [file cardio_v6i1e33286_app1.docx]

**SUPPLEMENTAL MATERIAL**

**Appendix 1**

Usability Survey

1. I know what kind of health issue I have.
   1. Strongly Disagree
   2. Somewhat Disagree
   3. Neither Agree nor Disagree
   4. Somewhat Agree
   5. Strongly Agree
2. I know the names of the medications I am taking.
   1. Strongly Disagree
   2. Somewhat Disagree
   3. Neither Agree nor Disagree
   4. Somewhat Agree
   5. Strongly Agree
3. I know why it is important to check my vital signs such as my weight, blood pressure, blood sugars, oxygen, etc.
   1. Strongly Disagree
   2. Somewhat Disagree
   3. Neither Agree nor Disagree
   4. Somewhat Agree
   5. Strongly Agree
4. I was comfortable using the new technology.
   1. Strongly Disagree
   2. Somewhat Disagree
   3. Neither Agree nor Disagree
   4. Somewhat Agree
   5. Strongly Agree
5. I did not worry about my privacy with the new technology that was used to keep track of my health.
   1. Strongly Disagree
   2. Somewhat Disagree
   3. Neither Agree nor Disagree
   4. Somewhat Agree
   5. Strongly Agree
6. Learning to take care of my health issue at home with new technology did not take too much time.
   1. Strongly Disagree
   2. Somewhat Disagree
   3. Neither Agree nor Disagree
   4. Somewhat Agree
   5. Strongly Agree
7. Overall, I am satisfied with my experience in the HF-SMART program.
   1. Strongly Disagree
   2. Somewhat Disagree
   3. Neither Agree nor Disagree
   4. Somewhat Agree
   5. Strongly Agree
8. I would recommend this program to others.
   1. Strongly Disagree
   2. Somewhat Disagree
   3. Neither Agree nor Disagree
   4. Somewhat Agree
   5. Strongly Agree
